# “Establishing healthy habits and lifestyles early is very important”: parental views of brain health literacy on dementia prevention in preschool and primary school children

**DOI:** 10.3389/fpubh.2024.1401806

**Published:** 2024-08-06

**Authors:** Tayla M. Chalhoub, Erin Mackenzie, Joyce Siette

**Affiliations:** ^1^The MARCS Institute for Brain, Behaviour and Development, Western Sydney University, Westmead, NSW, Australia; ^2^School of Education, Western Sydney University, Kingswood, NSW, Australia

**Keywords:** dementia, parents, barriers, acceptability, facilitators

## Abstract

**Introduction:**

Parents have the potential to drive healthy lifestyle behaviors through educational initiatives. This study aims to understand the prevalent thoughts and perceptions parents have toward brain health educational programs for preschool and primary school settings, whilst also contributing to a comprehensive understanding of the role parents can play in the broader context of dementia reduction strategies and the cultivation of brain health awareness among children.

**Methods:**

Parents with children aged between 2 and 11 years old were interviewed about their current knowledge of dementia, prior beliefs, current lifestyle factors and opinions on educating their children from a young age on the topic of dementia literacy. Thematic deductive analysis was employed to systematically categorize and interpret the qualitative data obtained from these interviews.

**Results:**

Thirty parents (*M*_age_ = 38.6, SD = 4.9, Range = 32–48) identified three core themes on *nurturing bodies and minds* (e.g., conceptualizing the link between intellectual engagement, continuous learning, and the prevention of cognitive decline), *brain health literacy* (e.g., current knowledge and awareness of brain health, dementia and associated stigma, and provision of age-appropriate health literacy) and *parental concerns* (i.e., barriers to initiating conversations about dementia with children and strategies to address and alleviate parental concerns). Parents possessing prior knowledge of dementia and its modifiable risk factors exhibited greater propensity to educate their children on the associated risk factors.

**Conclusion:**

Our study highlights the vital influence of parents’ experiences, health literacy, and education on the acceptance of brain health education for children. Future interventions should target stigma reduction, enhance awareness, and offer accessible information on modifiable dementia risk factors, enabling a conducive environment for active parental involvement in educating children about brain health and contributing to future well-being.

## 1 Introduction

In an age marked by an increasing interest in child development and well-being, coupled with the proliferation of information related to brain health, the role of parents as primary caregivers and decision-makers in shaping their children’s cognitive and emotional growth has become increasingly pivotal (1). Central to this dynamic is the concept of brain health literacy – the capacity to access, comprehend, evaluate, and utilize information pertaining to the brain’s development, functions, and potential influences on overall well-being, including the prevention, promotion, and management of brain-related conditions and diseases (2, 3).

Understanding brain health literacy is paramount in addressing neurocognitive disorders (4, 5). Contemporary research highlights the importance of proactive awareness and modification of 12 key modifiable risk factors to prevent or delay dementia (6). These factors include less education, hearing loss, hypertension, obesity, smoking, depression, social isolation, physical inactivity, diabetes, alcohol excess, traumatic brain injury, and air pollution (6). Early-life interventions starting from when a child becomes of age when they begin to learn habits, particularly through education, can empower children for informed decision-making regarding dementia-related risks in the future (7).

In the context of early-life interventions, education is considered a transformative force (8, 9). Livingston et al. (6) identified that early general education contributed to dementia risk reduction. Indeed, cognitive ability is positively correlated with educational attainment, although this growth reaches a plateau after adolescence (10). Policy considerations thus must prioritize childhood education as a key intervention against dementia prevalence.

However, recent studies have indicated low levels of brain health literacy among individuals worldwide (11–15). This lack of knowledge poses the risk of delayed diagnosis and potential obstacles in accessing timely medical care and support (14, 16, 17). Bradford (16) and Cahill (14) further emphasize the connection between limited awareness and delayed interventions due to ignorance of modifiable risk factors. On the other hand, research by Cations (15) and Lincoln (18) underlines the empowering potential of higher dementia literacy, enabling individuals to identify symptoms, mitigate risks, ensure timely diagnoses, and manage cognitive health proactively.

Current global policy responses focus on educating the older population of the modifiable risks of dementia (4). There is very limited information concerning public perceptions of risk and prevention on dementia in early years, meaning intervention strategies fail to take into consideration children’s and parents’ belief and experiences (19, 20). In particular, parents are a group uniquely positioned to influence not only their own well-being but also that of future generations, meaning that their perspectives are critical to consider.

Within this context, exploring the attitudes and beliefs of parents, and how these caregivers perceive the links between modifiable risk factors, can provide key strategies to effect behavioral change for the purpose of dementia risk reduction of the next generation. Targeting parents may therefore be a key strategy to achieve behavior change in adults and increase dementia literacy among their children. Targeting children during their preschool to primary school years presents a strategic approach for promoting behavior change in both their adult parents as well as increasing dementia literacy among children. This period is characterized by significant developmental milestones in children, wherein they are highly receptive to learning and influenced greatly by parental behaviors and attitudes (21). Firstly, parents serve as primary role models and educators for their children, shaping their beliefs, attitudes, and behaviors through direct interaction and observation (22). Secondly, by educating parents about dementia during these formative years, interventions can leverage parental influence to instill positive attitudes toward dementia and foster behaviors that support dementia literacy in children (23). Indeed, parents’ role in children’s health education is crucial; Bandura’s (24) Social Cognitive Theory emphasizes that parental adoption of health education relies on their own health behaviors and experiences. As such, the theory asserts parents as models, influencing children’s perceptions of the feasibility of certain lifestyle behaviors (24).

Furthermore, parental beliefs, attitudes, and self-efficacy significantly impact their ability to promote healthy behaviors within the family unit, enhancing the likelihood of children adopting similar lifestyle habits. The family environment during early childhood years provides a primary context for learning and socialization, enabling ongoing discussions and activities related to dementia within the family setting (25). Understanding parents’ perspectives on improving brain health literacy may contribute to formulating adequate preventative approaches to increase their children’s knowledge of the modifiable risks associated with dementia (20). Such conversations and introductions of dementia literacy concepts can also help normalize discussions about the condition and reduce stigma, laying a foundation for open communication within families (26).

The primary objective of this study is to therefore investigate parental perspectives concerning brain health education, specifically focusing on discerning prevalent thoughts and emerging trends in dementia literacy education for children of preschool to primary age. Our research question aims to identify how parents’ perceptions of brain health educational programs for preschool and primary school settings influence their understanding of dementia reduction strategies and the cultivation of brain health awareness in their children.

## 2 Methods

### 2.1 Participants

Participant demographic information can be seen in Table 1. Thirty participants (*M*_age_ = 38.6, SD = 4.9, Range = 32–48) were recruited through word of mouth, advertising on Facebook and an online participant database management system affiliated with the research group. The study included mostly females (27/30, 90%) with children ranging from 3 to 11 years old (*M*_age_ = 6.0, SD = 2.5).

**Table 1:** Sociodemographic characteristics of participants.

Variable	*n*	%	*M* (SD)
Age			38.6 (4.9)
Gender			
Female	27	90	
Male	3	10	
Marital status			
Married	27	90	
*De facto*	3	10	
Children			
1	16	53.3	
2	8	26.7	
3	5	16.7	
4	1	3.3	
Age of children			6 (2.5)
2	3	5.8	
3	7	13.7	
4	4	7.8	
5	9	17.6	
6	10	19.6	
7	3	5.8	
8	6	11.8	
9	3	5.8	
10	3	5.8	
11	3	5.8	
Child education level			
Pre-school	17	33.3	
Kindergarten	12	23.5	
Year 1	7	13.7	
Year 2	3	5.8	
Year 3	5	9.8	
Year 4	2	3.9	
Year 5	3	5.8	
Year 6	2	3.9	
Parents highest education			
High school	2	6.7	
TAFE	1	3.3	
Undergraduate/postgraduate	27	90	
Pre-existing health condition^a^	6	20	
Family health condition^b^			
High blood pressure	2	4.7	
Diabetes	7	16.3	
Cholesterol	2	4.7	
Dementia	11	25.6	
Heart disease	8	18.6	
Kidney failure	1	2.3	
Cancer	5	11.6	
Asthma	1	2.3	
Lupus	1	2.3	
Hepatitis	1	2.3	
Endometriosis	1	2.3	
Osteoporosis	1	2.3	
Mental health	2	4.7	

*N* = 30 (*n* = 1 for each condition). Participants were on average 38.6 years old (SD = 4.9), and participant age did not differ by condition.

a Reflects the number and percentage of participants answering “yes” to the question, “Do you have any pre–existing health conditions?”

b Reflects the number and percentage of participants answering “yes” to the question, “Do you have a family history of health conditions?”

### 2.2 Materials

#### 2.2.1 Demographics survey

The demographic survey consisted of nine questions about age, marital status, number of children, age of child/children, grade of child/children, highest education level, pre-existing health conditions and family history of health conditions.

#### 2.2.2 Semi-structured interview guide

The semi-structured interview guide consisted of 17 questions related to parents’ perspectives of brain health education for their children. The questions were derived theoretically from the existing literature (6) on adopting healthy behaviors, brain health interventions, parents’ role on child’s lifestyle and to gain insight into the research question. The interview questions were tested prior on a member of the research team to ensure validity and structure. The interview consisted of closed- and open-ended questions to identify focus responses and gather a better understanding of the respondents’ beliefs and attitudes.

### 2.3 Procedure

Recruitment commenced with researcher networks, word of mouth and online advertising (e.g., through Facebook). Interested participants were provided the study flyer, outlining what it means to be involved in the study, and if interested, to contact the research team to participate. Eligible applicants had to be aged over 18 years old, be a parent of a child/children of ages 3–11 and be able to provide consent. Parents who had younger aged children (e.g., <3 years were eligible to participant if they had a child aged >3 years as well. Participants expressed interest in the study by responding to the flyer and sending an email or message to the research team. Participants were then checked against the eligibility criteria and if eligible, were invited to an interview via Zoom. During the interview, they read though the participant information sheet, provided written and verbal consent and were then asked demographic questions (e.g., age, marital status, current number, and ages of children) (Table 1), followed by interview questions. Interview duration ranged between 20 and 50 min. Upon completion of the interview, participants were thanked for their time and reimbursed $20 through a e-gift card.

### 2.4 Data analysis

Interviews were recorded and transcribed using the live transcription software in Zoom. Transcripts were checked for accuracy and any identifying information was removed prior to analysis by the research team. Thematic deductive analysis was applied in accordance with the six steps outlined by Braun and Clarke (27) using NVivo (Version 12). Data familiarization, code development, theme identification, and validation were applied. Initial data codes were generated in relation to the aim of the study, for example, some of the codes included a focus on barriers (“children are too young”), education (“I need to be educated on dementia”) and solutions (“Needs to be interactive for children”). The codes were then sought through and sorted into potential themes and sub themes. The themes were then revisited where two researchers (JS, TC) coded the data independently and compared their findings together for similarities and differences. No discrepancies were evident, however if there were disagreements in the themes, a third party (EM) would have analyzed the data to resolve the discrepancy. Intercoder reliability was met as both coders agreed on how to code the same data.

## 3 Results

### 3.1 Themes

**Figure 1:**
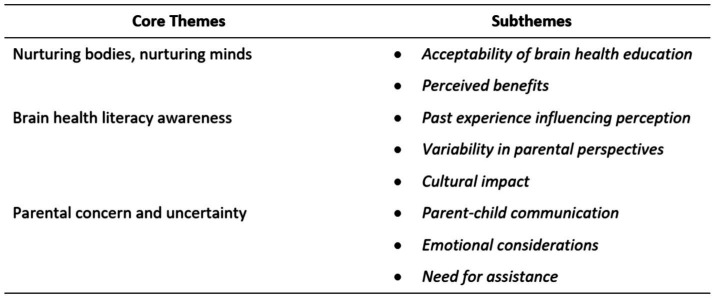
Core and subthemes.

Three core themes were identified, with a total of eight subthemes across the 30 participants (Figure 1).

### 3.2 Nurturing bodies and minds

Parents acknowledged the importance of taking care of both physical and mental well-being in children. Parents valued their children’s mental and cognitive well-being and believed it was important for them to learn about brain health.

#### 3.2.1 Acceptability of brain health education

Most parents recognized the importance of brain health education for their children’s overall development and future brain health. Parents who understood the importance of brain health education and who were well informed were more likely to be accepting of it, *“So, I think if we incorporate all these modifications, and if there is something that can help reduce the risk then why not”* and *“I mean, if he can avoid it in the future that will benefit him, so I guess [it’s] important.”* Parents who recognized the significance of early education, had firsthand experience with dementia’s effects, and grasped its relevance were more receptive to educating their children. This is important to note as it is critical healthy habits are established early to enhance a healthy lifelong term:

*“I have experienced dementia taking hold a lot quicker when you live in isolation through my parents… It is critical they understand this. People are only living longer and longer so dementia is a big topic now, and establishing healthy habits and lifestyles early is very important.”*

#### 3.2.2 Perceived benefits

Parents who were accepting of brain health education believed their children would benefit from such education, *“I think all the normal conversations around improving health through food and activity can feed into improving your mind and mental health.”* Some parents believed their children would benefit from being educated due to increasing their motivation*, “I think my daughter would be very concerned by this issue, and it might actually improve her to pay more attention and have an increased interest in staying active and eating healthy, as she currently lacks motivation.”* Whilst another parent saw the benefit of instilling habits at a younger age, *“With the young children, it’s really important to create a good habit of eating for them. So, when they grow up, they will stay the same, and that will prevent them from lots of diseases, including dementia as well.”* By instilling healthy habits early on, brain health education can help promote lifelong learning:

*“Yeah, I think for them it is being able to understand that early on setting up those good routines and good habits, especially if you know we are talking about, you know, the ability to, I guess, control those sort of things, then setting up those habits. And, you know, getting kids, you know, familiar with that early on can only benefit that.”*

The importance parents placed on their children learning about the link between physical activity, nutrition and brain function was highly recognized *“To be healthy is a good thing, and getting it into them when they are young, and getting them understanding how their body works. For the young is really helpful, long term.”*

### 3.3 Brain health literacy awareness

This theme identified how parents’ past health experiences played a role in their perception on brain health literacy and education for their children.

#### 3.3.1 Past experience influencing perception

Parents who have had positive experiences with health education in their own childhood or adulthood were more likely to have a positive perception of its importance for their children: *“Very comfortable, most of the risk reduction factors apply to other areas of life as well.”* Parents who had personally experienced health issues or witnessed someone with health issues such as dementia, showed to have had a heightened perception of the importance of brain health education. One parent stated, *“Yes, I work with the older adult so I have a lot of experience working with people with dementia”* and when asked *“How important is it for you that your children understand the importance of dementia risk reduction?”* they stated *“I think it is important, it is good for them to know the short term changes they can make to live healthy….”* Other parents reported when their own parents had experienced dementia, they wanted their children to learn the lifestyle habits early to reduce the risk of getting dementia later on in their life:

*“Both my parents had dementia, so I experienced it first hand through them… Yes, it is better for them to learn healthy habits when they are younger, than when they are older and it will be harder to break their unhealthy habits…Pretty important, because I want him to grow up and be healthy and not have to endure dementia. I want him to understand how to reduce dementia as much as possible.”*

#### 3.3.2 Variability in parental perspectives

This subtheme highlighted the diversity of viewpoints among parents regarding the importance of dementia education for their children. It indicated parents’ attitudes and priorities in this regard can vary significantly, potentially influenced by contextual factors, personal experiences, and their understanding of the topic.

Parents described the complex relationships between parental protection, age-appropriate education, and concerns about emotional well-being when considering dementia education for children. One parent identified the delicate balance parents must strike between raising awareness about important health issues like dementia and safeguarding their children’s emotional well-being, *“No, I think my children are too young… My concern is I would worry them about something they would never need to worry about, and just scaring them in general.”* Some parents were unsure of the benefits of educating their children at a younger age and connected their children’s understanding to personal experience:

*“Maybe starting from 8 or 9. When they have a bit more understanding. Maybe if they see it in their family like? If they get other grandparents, have it that kind of thing, then they might have a closer affinity to know what dementia is and how it affects people.”*

Parental perspective on the perceived importance of dementia education for children also indicated a degree of indifference and lack of urgency regarding this issue, with another parent stating, *“look to be completely honest, I would not say it’s particularly important right now.”*

#### 3.3.3 Cultural impact

Parents’ cultural background and generational differences can shape their perception of brain health education. Parents were asked if any specific cultural factors might influence how parents approach talking about dementia risk reduction with their children, *“I think certain cultures do not know much about dementia, and have a certain stigma about dementia, and so they would not discuss it with their children.”* In certain cultures, there may be stigma or taboos associated with dementia which can lead to parents being less willing to engage in conversations about brain health or provide formal education for their children:

*“I guess in my work I come across certain cultural backgrounds where it is just absolutely no way you admit that family member to hospital, any care facility despite them having quite profound dementia where it is affecting their own safety.”*

For some cultures, they may not place much importance on dementia because of limited exposure and knowledge:

*“Yes, I believe dementia probably wasn’t a big problem for parents that came from other countries where they were not around as much processed food and technology saturated environment, so they would not see dementia as a big issue, and therefore would not care about or teach lifestyle habits to reduce dementia.”*

### 3.4 Parental concern and uncertainty

Parents acknowledged the presence of existing internal and external sources of concern about their children becoming scared of the future and uncertainty toward their children being educated on brain health. The following theme highlighted how barriers of communication, emotional considerations and need for assistance hindered parent’s acceptance of brain health education for their children. Parents discussed three main considerations when providing their children with dementia literacy education. In general, parents identified the dynamics of parent–child communication, the emotional considerations involved in health education, and the need for further research or strategies to effectively address these concerns while ensuring that essential information is shared responsibly and empathetically.

#### 3.4.1 Parent–child communication

Parent–child communication was seen as a barrier for some parents in effectively educating their children on brain health. Parents struggled with navigating an understanding of how to educate their children effectively while also considering their emotional and mental well-being, *“My concern is I would worry them about something they would never need to worry about, and just scaring them in general.”* Some parents identified a delicate balance between providing children with valuable knowledge about health and risk factors while safeguarding their mental and emotional well-being and the need to filter out inappropriate or potentially harmful content, *“Children getting the wrong message. If you discuss this issue [dementia risk] too young, children may become obsessed with the issue and change their lives around it.”*

#### 3.4.2 Emotional considerations

Parents were concerned that children might misconstrue the information or misinterpret its significance, potentially leading to unnecessary worry or anxiety. This concern highlighted the importance of age-appropriate communication and emphasized the role of parents in making informed decisions about when and how to discuss certain topics with their children. Parents needed to weigh the benefits of knowledge against the potential negative consequences, *“I think you would not want to scare them too much, still would like them to live their life.”* Some parents commented on providing age-appropriate education that communicated the required messages in simplified language when discussing dementia. Parents reflected the need to make information accessible and comprehensible to support children’s understanding, engagement and knowledge transfer, *“I think it would be a matter of reducing the language like the complexity of dementia. In lowering the words used so that they can understand it at their level.”* This information needed to be aligned with principles of age-appropriate and adaptive communication, so that children at different age groups with varying levels of cognitive abilities and understanding could absorb the information readily. Parents also held diverse opinions regarding the timing of these conversations, believing that developmental readiness, age-appropriate education, and the availability of communication strategies would influence when the conversation should take place, *“I think around when they start school, because they probably have a better grasp of concepts. So probably around the 5 to 6 year old mark, I think,”* and *“Mid to later primary school once they understand social cues and social behaviors.”*

#### 3.4.3 Need for assistance

Many parents discussed their limited knowledge or education on the topic of dementia risk reduction. This lack of education and knowledge had the potential to hinder their ability to effectively educate their children about dementia risk reduction, *“My other concern is I do not feel educated enough myself with the issue of dementia to teach my kids”* and *“My own knowledge is a barrier.”*

Parents expressed their anxiety about providing potentially inadequate responses due to their limited understanding of the subject matter. Participants discussed the need for adequate information and resources to support effective communication and education within the parent–child dynamic, *“Not being able to answer their questions because I do not know much myself”* and *“I do not know enough myself to be able to teach them properly.”* Indeed, a recurring theme that emerged from participants’ responses was the key role of information access and parental education. Many parents expressed the need to access the right level of information to effectively convey the subject matter to their children. This connection between their own knowledge and their willingness to educate their children was highlighted, indicating that an increase in their understanding would lead to more open discussions*, “I feel I need access to the right level of information to effectively teach my children,”* and *“I guess if I was educated on it I would be more likely to kind of talk about it more.”*

Furthermore, participants identified various resources they found valuable in facilitating these discussions, including handouts, printouts, case studies, videos, and short educational materials. The desire to see examples of how other parents engage in these conversations demonstrated a need for practical guidance and insights*, “Maybe some tips, like some handouts, some printouts. Material basically that show how other people talk to their kids.”*

Moreover, participants realized the significance of awareness campaigns and educational materials targeting parents. They expressed a need for specific parenting websites and classes focusing on dementia risk reduction factors. These resources were seen as valuable tools for parental education and, in turn, effective communication with their children, *“Specific parenting websites that might target dementia”* and *“Classes teaching dementia risk reduction factors would be great for parents to learn, and just material for parents to be able to learn more, so websites. More ads to increase awareness.”* Some parents regarded school-based education as an ideal approach, citing the potential benefits of peer support and a structured environment for discussing these topics, *“Like, if it’s say in a school environment, there’d probably be more support, for you know they see other people as well [as] being involved.”*

## 4 Discussion

The current study aimed to investigate parents’ perceptions of brain health education for their children and to understand how current educational and familial practices could be used to build children’s brain health. As the primary caregiver in a child’s life, parents play a critical role in the way brain health literacy is educated and delivered (1). Whilst parents were keen to introduce the concept of brain health to their children, considerations of parent–child communication, emotional considerations and need for assistance were significant causes of parents’ reservations toward brain health education. This meant fewer parents were accepting of educating their children unless their needs for further education and awareness were met. Whilst acceptability and perceived benefits in educating their children were seen as an investment in their children’s cognitive and emotional development and well-being, some parents continued to voice concerns about the effectiveness and purpose of educating their children. This suggests that parents are aware of the benefits of brain health literacy, especially those who had prior knowledge of and experience with dementia.

Parental acceptance of brain health literacy and education appeared to be linked with the perception that such education could promote a healthy lifestyle for their children. Some parents, specifically those who had prior knowledge and education on a health condition, could see the benefits, which included their children instilling habits at a younger age whilst improving their physical and mental well-being to help them live a healthy life. A number of barriers to adoption, such as parent–child communication, emotional considerations and need for assistance, were identified. Parents believed brain health literacy would involve information which would not be age-appropriate for younger children. Parents considered the relevance and context of the information based on their child’s emotional readiness and whether the information was relevant to their child’s needs. This finding contributes to the existing literature, highlighting how a parent’s decision about delivering complex information is influenced by their perceptions of their child’s cognitive and emotional development (28–30).

While the provision of health education was acknowledged as important, a commonly identified barrier was the linkage of this information to parents’ own beliefs and prior observations and experiences with their children, aligning with the theoretical frameworks of Ajzen and Fishbein (31) and social learning theory (32). Both theoretical approaches highlight how mutual environmental and cognitive factors interact to influence human learning and behavior. Indeed, parents expressed the importance they placed on their children being educated at a young age if they deemed their child as being cognitively and emotionally ready. Parents’ past health observations and experiences aided in recognizing the repercussions of not introducing their children to brain health literacy. These findings align with the aforementioned framework by suggesting that individuals learn through observing and imitating others within their environment and that their beliefs are based on their own experiences which can determine whether they will use the knowledge and transform it into practice.

Parents expressed concerns about their limited knowledge or education regarding dementia risk reduction. This deficiency in understanding had the potential to impede their ability to effectively educate their children about dementia risk reduction. Indeed, prior research indicates that parents with a high level of knowledge about child development are more likely to provide age-appropriate books and learning materials tailored to their children’s interests, engaging in increased reading, conversation, and storytelling compared to parents with lower knowledge levels (33, 34). Furthermore, children with highly educated parents are more likely to exhibit health literacy and associated behaviors compared to children with less educated parents (35). Thus, parents with higher levels of health literacy may be more likely to engage in behaviors that promote their children’s health literacy and understanding of dementia risk reduction strategies. Conversely, parents with lower health literacy may struggle to impart this knowledge to their children, potentially leading to lower levels of health literacy among their offspring. This suggests that efforts to improve parental health literacy could have significant benefits in promoting children’s understanding of dementia risk reduction strategies and overall health literacy levels.

Parents articulated that cultural factors could potentially influence the way in which other parents perceive brain health literacy for their children. Supporting the results from Shaw (35), different cultures may have varying beliefs, attitudes and practices related to brain health, which can influence how parents perceive the need for brain health education. In certain cultures, there may be stigma or taboos associated with brain health, varying beliefs and priorities and language and communication barriers when it comes to brain health education.

Our study highlighted the significant influence of perceived benefits and a strong commitment to children’s health on parental behaviors within the context of health education. These findings are congruent with the Health Belief Model (36), which emphasizes the pivotal role of perceived benefits in shaping parental acceptance of brain health education for their children. Parents who discerned clear advantages in educating their children about brain health exhibited higher receptivity, aligning with the core principles of the model (36). This alignment is also consistent with prior research, indicating that perceived program benefits were linked to a greater intention to participate in parenting programs among both mothers and fathers (37). Additionally, parents who prioritized children’s well-being and engaged in healthy behaviors described greater openness to brain health education. This perspective resonates with the model’s emphasis on perceived susceptibility and severity, underlining those parents who perceive their children as vulnerable to health issues and consider these issues as significant are more likely to engage in health education. These insights align with prior studies which identified that parents who rated parenting programs as more beneficial were more likely to express interest in attending such programs (38, 39).

### 4.1 Implications and future directions

**Figure 2:**
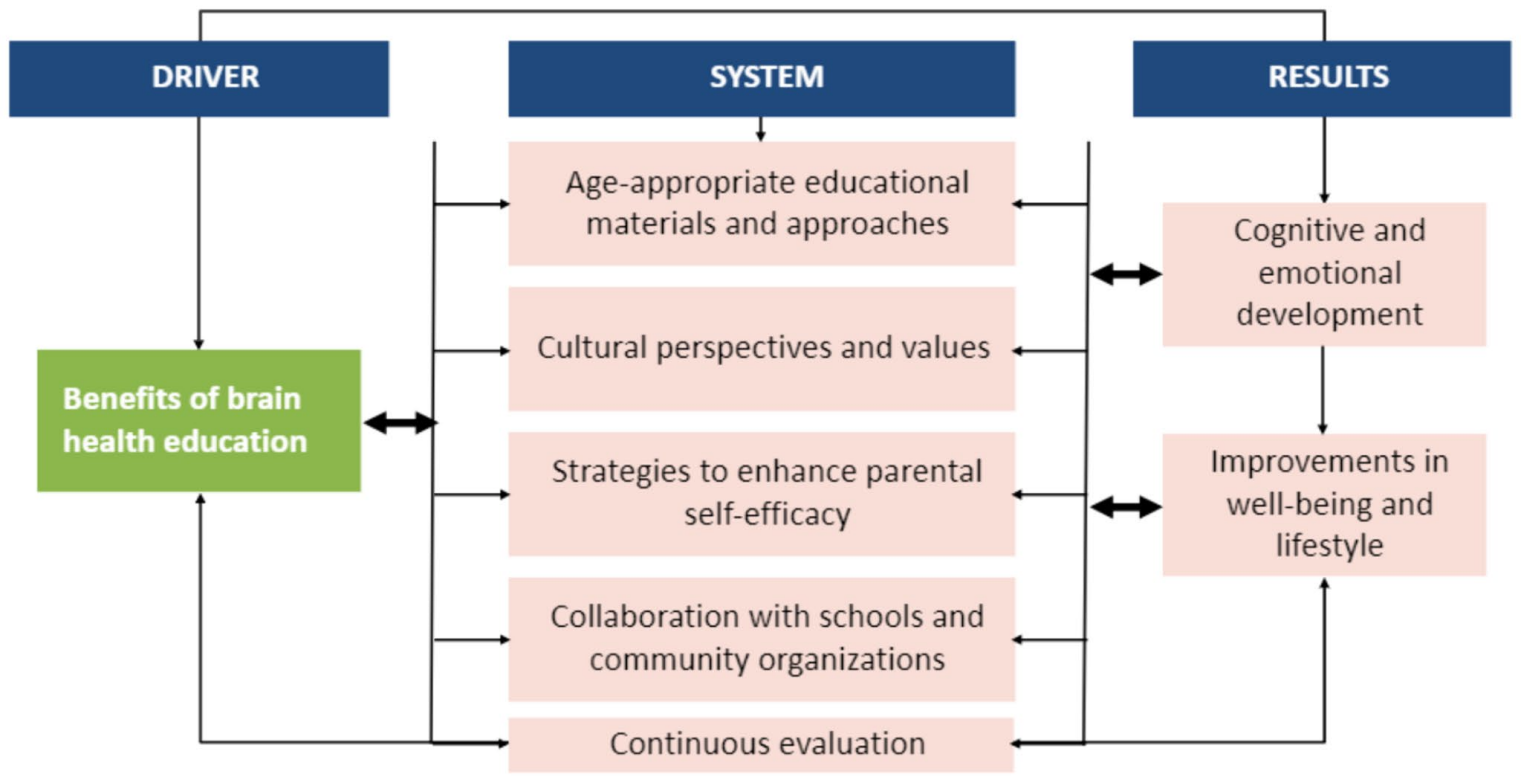
Proposed guide for the design, delivery, and implementation of brain health education programs.

A summary of the key educational brain health program aspects is provided in Figure 2. Such programs should ideally communicate the tangible benefits of brain health education to both parents and children. Recognizing that parents are more likely to engage in health-related behaviors when they perceive the benefits as outweighing the barriers, this component serves as a powerful motivator. By illustrating how brain health knowledge can positively impact children’s cognitive and emotional development, educators can also inspire parental engagement and commitment to the educational process.

We advocate for the incorporation of age-appropriate educational materials and approaches. This approach ensures that the content is engaging and comprehensible to children at different stages of development. Tailoring brain health education to align with developmental milestones is also likely to enhance the overall effectiveness of the program.

Addressing parents’ concerns about their own knowledge and confidence is crucial. To this end, future programs should incorporate strategies to enhance parental self-efficacy. These strategies may include providing parents with accessible resources, guidance on effective communication, and opportunities for parent education, so that parents could be empowered with the tools and confidence to educate their children. Current available tools to increase brain health education among children and parents include “My Brain Robbie.” “My Brain Robbie” is a more recent initiative designed to promote brain health amongst children aged 6–12 years. This campaign involves an animated video to help children learn about the eight steps to being brain healthy to fill a current educational gap. The “Healthy Brain Program” is another program which aims to address the prevalence of brain disorders such as dementia. The program involves exercises which parents are able to use with their children to promote recognition of risk reduction strategies and motivate changes to allow for the development of a healthy brain lifestyle.

Future research efforts should continue to encourage collaboration with schools and community organizations to reinforce brain health education, such as the “Healthy Brain Program” and “My Brain Robbie” to make them more well known. Addressing parents’ concerns about their own knowledge and confidence is important. Future programs could incorporate strategies to enhance parental self-efficacy by providing accessible resources, guidance on effective communication, and opportunities for parent education. Tools like “My Brain Robbie” and the “Healthy Brain Program” offer innovative approaches to promoting brain health among children and parents and should be tailored and leveraged to increase awareness and adoption.

Furthermore, individuals living in rural areas and from underserved populations may not align their priorities and concerns with include brain-related diseases (40, 41). As such, training or conversations about preventing diseases that are not recognized as priorities may not be well-received or valued in these communities. Future studies should aim to engage a more diverse group of participants to ensure a more inclusive range of viewpoints and consider the specific health priorities of different populations.

By creating a supportive environment where consistent messages about brain health are delivered both at home and in educational settings, the impact of these programs can be maximized. This collaborative approach creates a supportive environment where children receive consistent messages about brain health, both at home and in their educational settings. Education also needs to be delivered whilst acknowledging the diverse cultural backgrounds of families, whilst promoting and developing culturally sensitive materials and approaches. Brain health education should resonate with the cultural perspectives and values of parents and children, ensuring that the information remains relevant and relatable. Finally, to ensure the ongoing effectiveness of brain health education programs, future programs require continuous evaluation. Feedback from parents and children should be collected and used to refine materials and approaches. This iterative process can ensure that the education remains engaging and impactful.

### 4.2 Strengths and limitations

The present study exhibits several strengths and limitations that warrant consideration. One strength lies in an appropriate sample size for a qualitative study, which allowed for a comprehensive exploration of parents’ perspectives on brain health education. This sizeable participant pool facilitated the identification of diverse viewpoints, enriching the depth and breadth of the findings. However, the absence of fathers’ voices in the study raises gender imbalance concerns regarding the generalizability of the findings, as they may not fully capture the perspectives, experiences, and potential contributions of fathers in the context of brain health education. Another limitation of this study is the potential bias introduced by the participant selection process. While approximately 25% of the sample had a family history or affiliation with dementia, which likely heightened their interest in the topic, we may have inadvertently excluded individuals without such personal experiences or intrinsic interest. This limitation suggests that our findings might not fully represent the perspectives of the broader population who are less familiar with or concerned about dementia. Furthermore, the study encompassed a range of opinions on potential cultural barriers, resulting in diverse perspectives. This diversity of viewpoints makes it challenging to extrapolate the study’s conclusions to specific cultures and the associated barriers. Future research should consider seeking greater paternal, geographical, socioeconomic, spiritual, linguistic and cultural representation, employing targeted cross-cultural studies, and exploring alternative data collection methods. Additionally, investigating the long-term impact of brain health education on children’s cognitive and emotional development and well-being could provide alternative insights for further enhancing educational interventions.

## 5 Conclusion

Brain health literacy was deemed crucial and widely embraced by most parents. Notably, when parents had awareness of the potential consequences and held belief in the modifiable risk factors associated with dementia, they were more inclined to take proactive actions. However, some parents remain restricted to accepting and participating in brain health education due to the connection between their prior beliefs, culture and lack of knowledge and education. Well-rounded programs are needed to address language barriers, cultural barriers, and low health literacy simultaneously in prevention settings. Our results offer fresh perspectives on parental views regarding the significance of dementia education for children and highlight the necessity for additional investigation into parental attitudes and the contextual influences shaping their perceptions. This knowledge will help develop more comprehensive educational strategies in the future.

## Data availability statement

The datasets presented in this article are not readily available because data is potentially identifiable. Requests to access the datasets should be directed to JS, joyce.siette@westernsydney.edu.au.

## Ethics statement

The studies involving humans were approved by the Western Sydney University Human Research Ethics Committee. The studies were conducted in accordance with the local legislation and institutional requirements. The participants provided their written informed consent to participate in this study.

## Author contributions

TC: Data curation, Formal analysis, Visualization, Writing – original draft, Writing – review & editing. EM: Supervision, Validation, Writing – original draft, Writing – review & editing. JS: Conceptualization, Data curation, Formal analysis, Investigation, Project administration, Resources, Supervision, Validation, Visualization, Writing – original draft, Writing – review & editing.
